# Unravelling the mystery of the central dentinogenic ghost cell tumor- a rare case report and recurrent insights

**DOI:** 10.4322/acr.2024.510

**Published:** 2024-08-15

**Authors:** Jagveer Yadav, Mala Kamboj, Anju Devi, Anjali Narwal, Deepti Chhikara, Bhawna Saini

**Affiliations:** 1 Department of Oral Maxillofacial Pathology and Microbiology, Post Graduate Institute of Dental Sciences, Pandit Bhagwat Dayal Sharma University of Health Sciences, Rohtak, Haryana, India; 2 Department of Oral Maxillofacial Surgery, Post Graduate Institute of Dental Sciences, Pandit Bhagwat Dayal Sharma University of Health Sciences, Rohtak, Haryana, India; 3 Department of Oral Medicine and Radiology, Post Graduate Institute of Dental Sciences, Pandit Bhagwat Dayal Sharma University of Health Sciences, Rohtak, Haryana, India

**Keywords:** Maxillary Neoplasms, Odontogenic Cyst, Calcifying, Odontogenic Tumors

## Abstract

Dentinogenic ghost cell tumor (DGCT) is a rare benign neoplasm form of calcifying odontogenic cyst (COC) characterized by ghost cells. Although benign, it presents an aggressive behavior. DGCT accounts for 2% to 14% of all COCs and less than 0.5% of all odontogenic tumors. It is a benign odontogenic tumor despite its local invasion and the likelihood of recurrence. To detect recurrence, central DGCT patients must be monitored long-term. We present the case of a 51-year-old male who reported pain in the right upper back tooth region. On examination, a soft to firm, bright red swelling was present in the buccal vestibule and gingival margin of the maxillary right first and second molar, which extended up to the palate. Histopathological analysis confirmed the diagnosis of a DGCT, which occurred in a previously treated calcifying odontogenic cyst. The case is reported here, along with a review of the literature update of such recurred instances in the past.

## INTRODUCTION

Dentinogenic ghost cell tumor (DGCT) has been regarded as the solid, neoplastic presentation of a calcifying odontogenic cyst (COC), as stated by Praetorious et al.^1-3^ in 1981. It is an uncommon type of odontogenic neoplasm that is locally invasive and accounts for only 2-14% of all COCs. However, in the current edition of the World Health Organization (WHO) classification of Head and Neck tumors (2017), DGCT has been classified under tumors of mixed epithelial-mesenchymal origin.^3,4^ It is primarily seen in the middle-aged and can present as intraosseous (central) or extraosseous (peripheral) neoplasms. Central ones are more aggressive, require careful monitoring, and must be treated with aggressive local resection to prevent recurrence.^5^

Both variants of DGCT predominantly occur in the mandible with a gender preference for males with a ratio of 3:2. Peripheral DGCTs are smaller than the central ones, with a higher prevalence in elderly patients. Ameloblastoma-like basal cell layer and ghost cells appear in islands of epithelial cells, which form a solid neoplastic growth in DGCT. These lesions can be challenging to diagnose clinically, given the complex nature of their appearance.^6^ This paper aims to describe a case of central DGCT that occurred in a previously treated COC, along with an updated literature review.

## CASE REPORT

**Figure 1:**
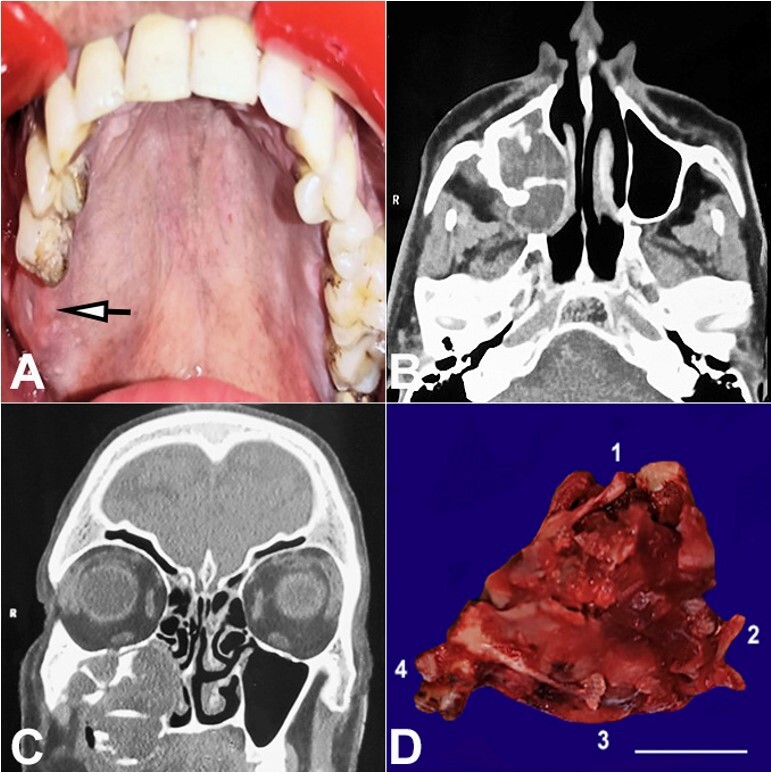
**A –** Intraoral photograph reveals soft to firm swelling in the vestibular region concerning right maxillary first and second molar extending till palate; **B –** CT axial view reveals an expansile lobulated soft tissue lesion bulging into maxillary sinus causing cortical destruction; **C –** CT coronal view reveals areas of amorphous calcifications, **D –** Excised gross specimen revealing tumor tissue morphology (scale bar = 2 cm) [1- lateral aspect of the maxillary first molar, 2-anterolateral wall of the maxilla and maxillary sinus, 3-palatal shelf and 4- a posterolateral wall of maxilla after pterygoid disjunction].

A fifty-one-year-old male patient reported to the dental college with a complaint of pain in the right upper back tooth region. History dates back to two months when he started to develop a change in color and swelling on the right side of the maxillary gingiva, which showed a gradual increase in size. The patient gave a previous history of surgery seven years ago for COC. On examination, a soft to firm, bright red, tender on palpation swelling was present in the buccal vestibule and gingival margin of the right maxillary first and second molar, which extended to the associated palate (Figure 1A).

Radiological investigations were carried out with a computed tomography scan of the paranasal sinuses that revealed an expansile lobulated soft tissue density extending from the right maxillary alveolus bulging into the maxillary sinus, right masticator and buccal space and into the nasal cavity. The lesion caused cortical destruction medial, postero, and laterally, extended into the hard palate, showing amorphous calcifications (Figures 1B and 1C). A provisional diagnosis of recurrent benign odontogenic cyst was made, considering the history of COC. An incisional biopsy was performed, and the tissue was sent for histopathological examination.

**Figure 2:**
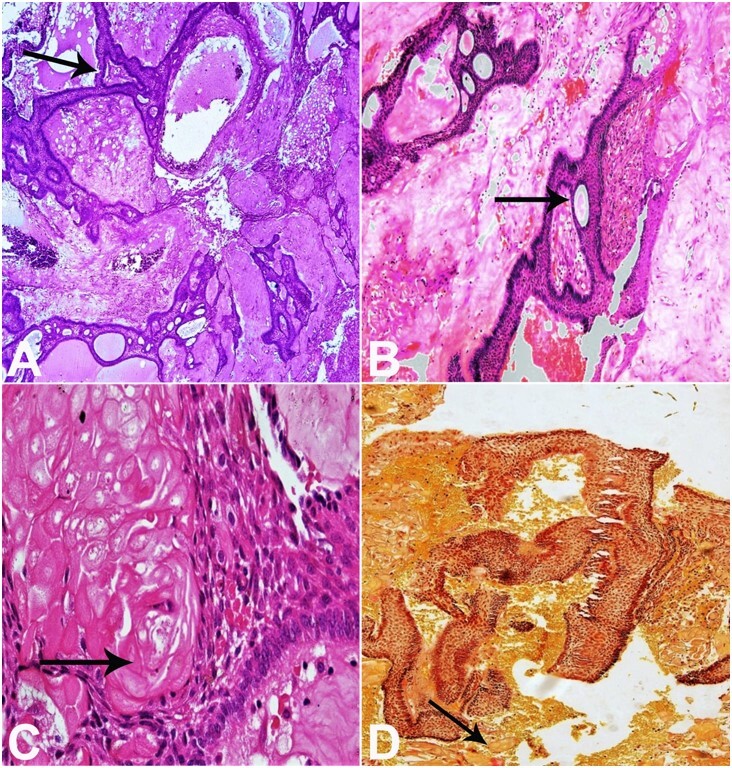
Photomicrographs depict histopathologic findings of DGCT. **A –** Ameloblastomatous epithelial proliferation - black arrow (H&E, 4X); **B –** Tall columnar ameloblast-like cells with reversal of polarity and ghost cells- black arrow (H&E, 10X); **C –** Dentinoid material - black arrow (H&E, 10X); **D –** Ghost cells stained yellow and dentinoid stained amorphous reddish pink - black arrow (Van Gieson, 4X).

On histologic examination, a plexus of odontogenic epithelium with tall outer columnar ameloblast-like cells and inner stellate reticulum-like cells were seen in a mature connective tissue stroma. The stroma showed the presence of abundant ghost cells and areas of dentinoid formation. A few foci of basophilic calcifications and bony trabeculae were also evident. On Van Gieson staining, the dentinoid material appeared reddish pink, and ghost cells took up yellow (Figure 2). Based on these findings, a diagnosis of DGCT was made. After the diagnosis, a surgical enucleation (Figure 1 D) was performed involving the anterior maxilla and nasal wall. The final diagnosis of DGCT was confirmed.

## DISCUSSION

According to the recent WHO classification, COC has been classified into three types, namely calcifying cystic odontogenic tumor (CCOT), dentinogenic ghost cell tumor, and odontogenic ghost cell carcinoma (GCOC).^2^ It is still unknown whether the surface epithelium or cell rests of Serre are responsible for the histogenetic origin of DGCT. Due to an incorrect differentiation process regulated by the WNT signaling system, missense mutation on codon 3 showed that catenin plays a significant role in its carcinogenesis.^5^ COCs account for 1-2% of all odontogenic lesions, of which 11.5% are represented as solid tumors, mainly the DGCT, while the remaining 88.5% are cystic.

Intraosseous DGCTs have been reported to occur in a vast age group of 8-80 years with slight male predilection with more propensity in the mandible than the maxilla. They are more aggressive, with a highly infiltrative growth pattern, and can recur even after resection; however, the extraosseous types are less common, chiefly occur in the sixth decade, arise in the alveolar mucosa or gingiva, and exhibit limited growth potential. Both variants show similar histopathological features.^2^ The present case of DGCT was in sync with the previous literature on DGCT, as it was found in a 51-year-old male with swelling in the maxilla.

Radiographically, DGCT presents as unilocular or multilocular radiolucency with scattered radio opacities resembling calcifications. Occlusal radiographs highlight its bicortical expansile nature, while in some cases, the presence of impacted teeth, as well as their root resorption, displacement, and root resorption of adjacent teeth, are also evident.^7,8^ Some of the lesions also show partially or ill-defined borders, and maxillary lesions even extend up to the orbital floor.^9^ The present case showed a similar radiographic pattern of amorphous calcifications and cortical destruction with invasion into the masticator and buccal spaces extending medially into the hard palate and nasal cavity, depicting its local aggressiveness.

The histopathology of a DGCT has a biphasic morphology, with predominant ameloblastomatous proliferation and a less prominent part of basaloid to stellate reticulum-like cells. The tumor also shows the presence of aberrant keratinization in the form of ghost cells and dentinoid or osteodentin-like material.^10^ The odontogenic epithelium is generally in the form of sheets and islands with ameloblastic differentiation, that occasionally undergoes cystic degeneration in a mature fibrous connective tissue. The ghost cells appear as larger, ellipsoidal, eosinophilic epithelial cells that have lost their nuclei and frequently calcify. If calcified, they are evident as small basophilic granules or coarse masses scattered throughout the odontogenic epithelium or stroma. Ghost cells are believed to result from secondary calcifications from squamous metaplasia with ischemia, apoptosis, aberrant keratinization, and coagulative necrosis. Juxtaepithelially, dysplastic dentin or dentinoid, which appears as an amorphous mass of eosinophilic material, including widely spaced cell bodies, is evident. Dentinoid material is thought to arise as a result of the body's inflammatory response to a large number of ghost cells, or probably, their large number triggers the formation of granulation tissue to lay down juxta epithelial osteoid that may calcify. On the other hand, it was also speculated that it might represent a metaplastic change in the connective tissue or could be an inductive effect.^5^ The epithelial cells are positive for CK 5,7,14 and 19, with a low proliferation index expressed by Ki67 <5%. Variable expressions of CD 138 and MMP9 have also been reported, which needs to be established further to shed light on the locally invasive nature and biological behavior of DGCT.^10^

Prompt diagnosis of DGCT is thus pertinent for a better patient prognosis, as the treatment plan is generally different for the two types of DGCT because of their variable rates of recurrence and malignization potential. Intraosseous lesions usually need a block excision or segmental resection with adequate safety margins, especially in poorly defined DGCTs.^8^ Depending on their size or extension, intraosseous DGCT has a high rate of local recurrence if treated with limited local resection or conservative therapy, while the extraosseous ones are usually treated by conservative local excision.

Intraosseous tumors seem to have the same chance of recurrence as conventional ameloblastoma, and most cases recur between 5 to 8 years after the first treatment. Unlike in the case of extraosseous variants, no case has been reported to recur. Sun et al. reported seven cases of intraosseous DGCT. They observed that five of them had tumor recurrence after being treated conservatively, while two patients had an aggressive surgical resection, and the tumor did not recur.^11^ After a literature search on such recurrences, we found fourteen intraosseous cases of DGCT that had recurred, including the present case (Table 1).

**Table 1:** Details of the previously recurred cases of Dentinogenic Ghost Cell Tumor along with the present case

**Ref**	**No. of cases**	**Age & Sex**	**Site**	**Radiographic Findings**	**Treatment**	**Diagnosis before recurrence**	**No. of Recurrences** **(Period of recurrence)**	**Diagnosis after recurrence**
^1^	5	37/M	LMx	Bone expansion, cortical bone resorption	Local curettage	DGCT	2 (4&1.5y)	DGCT
		43/M	RM	NA	Curettage, local resection, segmental resection	DGCT	4 (past 6 y)	DGCT
		42/F	RMx	Mixed radiolucent/radiopaque	Local curettage	DGCT	2 (3&2 y)	DGCT
		29/M	LM	Irregular lesion borders, calcified lumps	Local curettage	DGCT	2 (4&2 y)	DGCT
		36/M	LM	NA	Local curettage	DGCT	1 (3 y)	DGCT
^3^	1	42/M	LMx	Unilocular radiolucency	Enucleation	Unicystic ameloblastoma	1 (3 y)	DGCT
^12^	1	33/M	RPM	Well defined multilocular radiolucency, associated with an impacted third molar	Segmental mandibulectomy	Simple ameloblastoma	1 (4.5 y)	DGCT
^13^	1	48/M	LMx	CT showed a locally infiltrative mass	Left medial maxillectomy	COC/PDSCC	1 (1y)	DGCT
^14^	1	59/M	RPM	Unilocular radiolucent-radiopaque lesion	Segmental resection of the mandible	DGCT	1 (5 y)	DGCT
^15^	1	20/M	RPM	Multilocular radiolucency with tiny radiopaque materials	Enucleation	DGCT	2 (10&8 y)	DGCT
	1	21/F	RPM	Multilocular radiolucency with radiopaque materials	Enucleation	DGCT	2 (10&20 y)	DGCT
^16^	1	25/F	LM	A well-defined multilocular radiolucent lesion	Partial mandibulectomy	DGCT	1 (2 y)	DGCT
^17^	1	27/M	RMx	CT scan: large, rounded, cystic structure extending into max antrum	Enucleation	COC	2 (6w & 3m)	DGCT
PC	1	51/M	RMx	Cortical destruction, amorphous calcification	Enucleation	COC	1 (7 y)	DGCT

COC-Calcifying odontogenic cyst, CT scan-Computed tomography scan, DGCT-Dentinogenic ghost cell tumor, F-Female, NA-Not available, LM- Left Mandible, LMx- Left Maxilla, M-Male, PC- Present case, PDSCC-Poorly differentiated squamous cell carcinoma, RPM- Right Posterior Mandible, RMx – Right Maxilla, y-years, m-months, w-weeks

Ten cases out of these fourteen were reported in males while four were reported in females, with a mean age of 36.6 years. The mandible was affected in eight, while the maxilla was affected in six cases of intraosseous DGCT. Out of the 14 cases, five had been treated by enucleation, while four were treated by curettage and the other four by segmental resection, either mandibulectomy or maxillectomy. One case that recurred four times had been treated by curettage and segmental resection.

Central DGCTs can turn malignant and become odontogenic ghost cell carcinomas in sporadic cases. The present case underwent surgical enucleation and is currently being monitored. There have been no signs of recurrence, and the patient is currently symptom-free for the past eleven months.

## CONCLUSION

DGCT is a rare odontogenic tumor with unique histological traits and behaves aggressively. It is important to distinguish this lesion from other odontogenic neoplasms for the correct treatment based on their histology. DGCTs also present with unusual histological features, so keeping patients under regular follow-up is necessary. Moreover, central DGCTs frequently recur, so they need to be handled cautiously. So, it is mandated that central DGCTs be diagnosed early and treated aggressively to prevent recurrence.
